# Carcass detection and consumption by facultative scavengers in forest ecosystem highlights the value of their ecosystem services

**DOI:** 10.1038/s41598-022-20465-4

**Published:** 2022-09-30

**Authors:** Akino Inagaki, Maximilian L. Allen, Tetsuya Maruyama, Koji Yamazaki, Kahoko Tochigi, Tomoko Naganuma, Shinsuke Koike

**Affiliations:** 1 United Graduate School of Agricultural Science, Tokyo University of Agriculture and Technology, 3-5-8 Saiwai-Cho, Fuchu, Tokyo 183-8509 Japan; 2 Illinois Natural History Survey, University of Illinois, Champaign, USA; 3 Nature Preservation Division, Tochigi Prefecture, Utsunomiya, Tochigi Japan; 4 Department of Forest Science, Tokyo University of Agriculture, Setagaya, Tokyo Japan; 5 National Agriculture and Food Research Organization, Tsukuba, Ibaraki Japan; 6 Institute of Global Innovation Research, Tokyo University of Agriculture and Technology, Fuchu, Japan; 7 Institute of Agriculture, Tokyo University of Agriculture and Technology, Fuchu, Japan

**Keywords:** Ecology, Community ecology, Ecosystem services, Forest ecology

## Abstract

Scavenging is a common feeding behavior that provides ecosystem services by removing potentially infectious waste from the landscape. The importance of facultative scavenging is often overlooked, but likely becomes especially important in ecosystems without obligate scavengers. Here, we investigated the ecological function of vertebrate facultative scavengers in removing ungulate carcasses from Japanese forests that lack obligate scavengers. We found that mammals detected carcasses first more often than birds, and that raccoon dogs (*Nyctereutes procyonoides*) were the most frequent scavenger to first detect carcasses. However, we found no evidence of there being species that signal the location of carrion to other species via social cues. Instead, higher temperatures promoted earlier detection of the carcasses by scavengers, likely related to increased olfactory signals. The carcasses were completely consumed on average in 7.0 days, reasonably similar to other systems regardless of habitat, indicating that facultative scavengers are providing ecosystem services. Larger carcasses tended to take longer to deplete, but carcasses were consumed faster in warmer temperatures. Our results indicate that facultative scavengers were capable of consuming carrion and contributing ecosystem services in a forest ecosystem that lacks obligate scavengers.

## Introduction

Animals who scavenge provide structure and important ecosystem services to ecological communities^1–3^. For example, scavengers provide ecosystem services by removing carcasses that are a source of harmful pathogens from ecosystems^1,4,5^. These scavenger species range from invertebrates to vertebrates, each of which compete for ephemeral carrion^1,3^. Scavenger species are often diverse with different traits and can fill many different ecological roles within communities.

Within the vertebrate scavenger community, obligate scavengers (species, like vultures, that are totally dependent on carrion^6^) are key species in scavenging networks that make critical contributions to its ecological function^7^. Obligate scavengers are excellent at detecting carrion, as a result, lead to carcasses being consumed faster^8,9^. Specifically, while obligate scavengers are highly efficient at consuming carrion, they also promote the interspecific interactions with other scavengers by signaling carcass locations while also opening large carcasses and allowing other scavengers access, thereby enhancing the carcass consumption rate^6,10,11^. But scavenging by facultative scavengers (species that opportunistically feed on carrion such as carnivora and raptors^12^) is also widespread, even in the absence of vultures (e.g., Refs.^13,14^). Facultative scavengers also include some key species, with high olfactory acuity and social foragers, playing an important ecological role in the facilitation of carrion consumption in a scavenger community^11^. Thus, it is important to understand ecosystem function (i.e., carrion consumption) by the facultative scavenger community in a system where obligate scavengers are absent. Nevertheless, there are still limited studies evaluating vertebrate facultative scavenger communities (e.g., Refs.^15,16^).

Japanese forest ecosystem lacks obligate scavengers (i.e., vultures), and instead the scavenger community is composed of facultative scavengers, dominated by raccoon dogs (*Nyctereutes procyonoides*) and Asian black bears (*Ursus thibetanus*) that are the primary scavengers of large ungulate carcass such as sika deer (*Cervus nippon*; hereafter “deer”)^17,18^. Therefore, facultative scavengers could be especially important to ecosystem services in this system through removing carrion^16,19,20^. On the other hand, invertebrates are also active in this system except in winter. Vertebrates and invertebrates are known to compete for carrion especially warm temperatures^21,22^, and invertebrates may perform functional compensation of vertebrates, as evidenced by the consumption of house mouse *Mus musculus* (Muridae) in the insular system^23^. It is unclear whether facultative vertebrate scavengers contribute to the removal of deer carcasses in addition to invertebrates, and or whether there are species in the system (similar to vultures) that signal carcass locations to other scavengers. To understand the ecological role of vertebrate scavenging, it is important to evaluate how facultative scavengers detect and consume animal carcasses and consequently contribute to the removal of these carcasses.

Our objectives are to determine the scavenging patterns in (1) the detection time (the elapsed time between the placement of the carcass and the arrival of the first scavenger) and (2) the depletion time (the elapsed time between carcass placement and its complete consumption) of deer carcasses in a Japanese forest ecosystem, and to evaluate the factors that affect these scavenging patterns. We tested the following hypotheses:

- *Pattern of carcass detection time.* We hypothesized that the carcass detection time would differ between mammals and birds, as well as among vertebrate scavenger species depending on their ecological traits. We predicted that mammalian scavengers would detect carcasses first more frequently than avian scavengers due to the limited aerial visual aspects in closed canopy forests (see details in “Materials and methods” section), and specifically that raccoon dogs and Asian black bears would detect carcasses first more frequently than other scavengers due to their more frequent use of carrion^17^.
- *Key species signaling the carcass location to other species*. We hypothesized the presence of at least one key species that would signal the presence of carcasses to other scavengers. We predicted that this species detects carcasses first, and that carcasses detected by the key species leads to faster detections for other scavengers.
- *Factors affecting the carcass detection time for the scavenging community*. We hypothesized that carcass detection time would be affected by olfactory and visual cues. We predicted that first detection time for each carcass would be different based on temperature and the presence of understory vegetation (Table 1).
- *Pattern of the carcass depletion time*. We hypothesized that the carcass depletion time would be long due to the absence of obligate scavengers and given closed canopy forests cause delays in detection and consumption by avian scavengers. We predicted that the carcass depletion time would be one of the longest reported in the literature (e.g., > 21 days^18^).
- *Factors affecting the carcass depletion time for the scavenging community*. We hypothesized that the carcass depletion time would be affected by the size of the carcass and competition with invertebrate scavenging. We predicted that carcass depletion time would be negatively affected by carcass weight and positively affected by temperature (Table 1).

**Table 1:** A list of the factors that we tested for carcass detection time and carcass depletion time, including the variables and reasoning for each factor.

Factor	Variable	Reason	References
**Carcass detection time**			
Visual	The presence of understory vegetation	The absence of understory vegetation will make it easier for scavengers to detect carcasses	^28,29,40^
Olfaction	Temperature	The warmer temperatures will promote carrion stench due to increasing decomposer activity and lead to faster detections	^1,25^
**Carcass depletion time**			
Carcass size	Carcass weight	Larger carcasses will take longer to consume	^29,41^
Competition with invertebrate	Temperature	Invertebrate scavengers will be more active and consume more carrion in warmer temperatures	^22,42^

## Results

### Detection of carcasses

The mean detection time of mammalian and avian scavengers were 4.9 days ± 4.2 SD and 4.7 days ± 3.7 SD respectively. Mammalian scavengers detected most carcasses first (88.6%), and significantly more often than avian scavengers (11.4%, p < 0.001; Table 2). Raccoon dogs most often detected the carcasses first (40.9%), significantly more frequent than other species (p < 0.001; Table 2), partially supporting hypothesis 1a. Masked palm civets (*Paguma larvata*) and black kites (*Milvus migrans*) never detected carcasses first, which was significantly less than other species (p_masked palm civet_ = 0.009, p_black kite_ = 0.009; Table 2). Raccoon dogs also had the fastest mean detection time (3.3 d ± 3.5 SD, p = 0.004; Table 2). In contrast, masked palm civets (9.8 days ± 4.8 SE, p = 0.004; Table 2) had the slowest mean detection time.

**Table 2:** The percentage of first detected carcass and the mean of detection time ± standard deviation (SD) by each scavenger. The p-values show the results of Fisher’s exact tests (the former) and Wilcoxon rank sum tests (the latter) between each scavenger with all other scavengers. Significant values are in bold. The asterisks show two species (raccoon dog and red fox) visited first and at the same time in one carcass.

Common name	Species	First detection			Detection time ± SD (day)		
		Number	(%)	*p*	Mean ± SD (day)	n	*p*
**Mammal**		39	(88.6)				
Asian black bear	*Ursus thibetanus*	8	(18.2)	0.127	4.9 ± 3.8	32	0.818
Wild boar	*Sus scrofa*	3	(6.8)	0.450	6.4 ± 4.4	21	0.083
Raccoon dog	*Nyctereutes procyonoides*	18*	(40.9)	** < 0.001**	3.3 ± 3.5	37	**0.004**
Red fox	*Vulpes vulpes*	2*	(4.5)	0.203	5.0 ± 3.7	19	0.530
Japanese marten	*Martes melampus*	9	(20.5)	0.070	4.0 ± 4.3	19	0.129
Masked palm civet	*Paguma larvata*	0	(0.0)	**0.009**	9.8 ± 4.8	8	**0.004**
**Avian**		5	(11.4)				
Mountain hawk-eagle	*Nisaetus nipalensis*	2	(4.5)	0.203	5.6 ± 5.8	5	1.000
Black kite	*Milvus migrans*	0	(0.0)	**0.009**	6.4 ± 2.6	3	0.079
Jungle crow	*Corvus macrorhynchos*	3	(6.8)	0.450	4.2 ± 2.8	18	0.816

**Figure 1:**
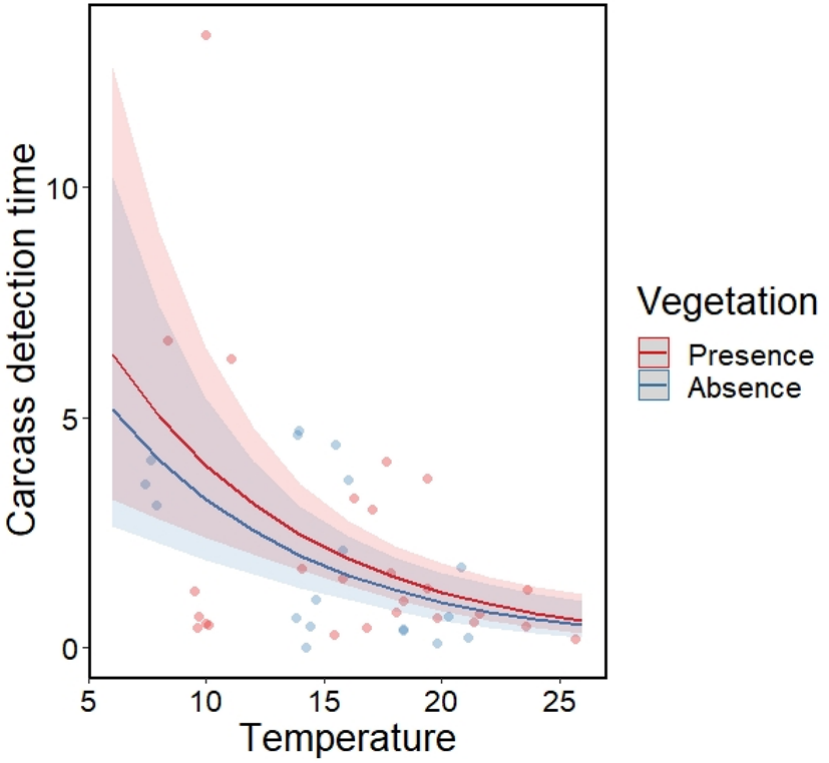
The predicted effects of temperature and understory vegetation on the detection time for ungulate carcasses in a GLM. Points represent measured values, lines represent mean estimates, and shaded regions represent 95% confidence intervals. We calculated the relationships while keeping other independent variables constant (temperature is set to the mean, understory vegetation is set to the reference level).

We considered whether raccoon dogs could be a key species for carcass detection, and raccoon dogs detecting carcasses first did not significantly affect the detection times for other scavengers (β = 0.266; p < 0.017), not supporting hypothesis 1b. Meanwhile, the first detection times for carcasses were significantly decreased by temperature (β = − 0.119; p < 0.001) but not influenced by the presence of understory vegetation (β = − 0.204; p = 0.472, Fig. 1), partially supporting hypothesis 1c.

### Carcass depletion time

**Figure 2:**
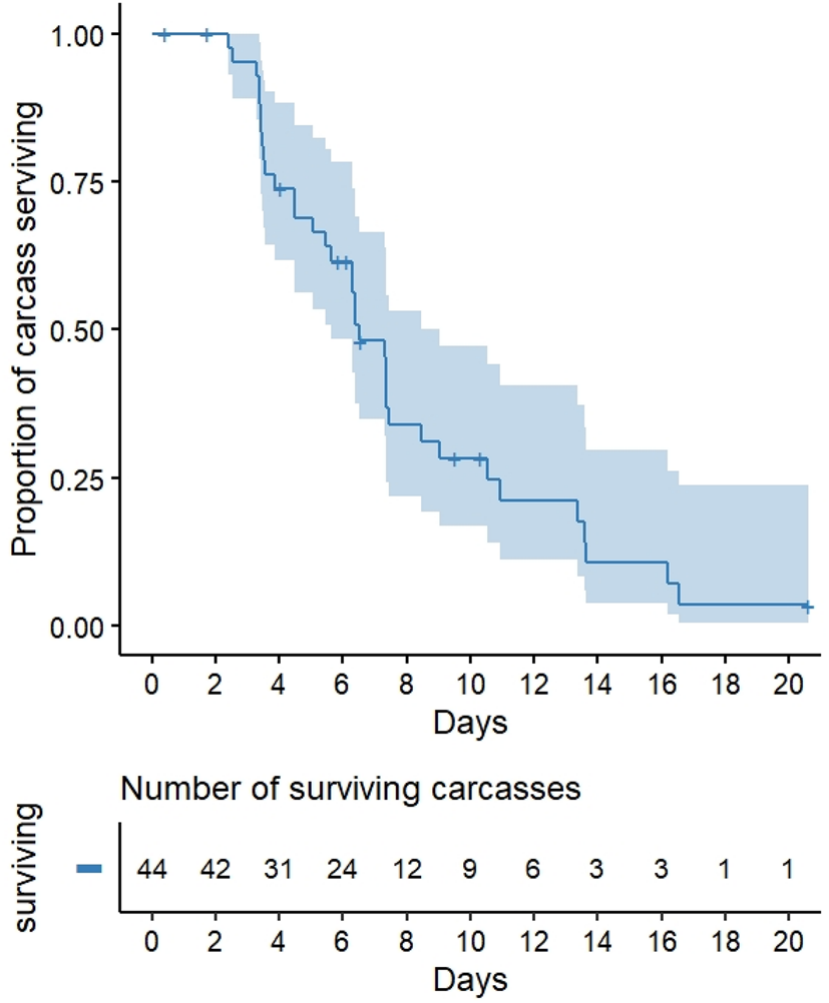
The probability of deer carcass persistence (n = 44) with corresponding 95% confidence intervals. The cross key shows the censored point in which carcass consumption had not been fully observed (n = 9).

The mean carcass depletion time was 7.0 days, and ranged from 2.4 to 20.6 days. The survival analysis showed that 97% of carcasses survived to 2.4 days and 96% of carcasses were completely consumed within 16.5 days (Fig. 2). This carcass depletion time ranks 18th of 26 studies reviewed in the literature comparison (Ref.^18^; Fig. S1). But our study was the second fastest depletion time among seven studies without obligate scavengers, and also 8th fastest depletion time among 15 studies conducted primarily in forests (Fig. S1), failing to support hypothesis 2a.

**Figure 3:**
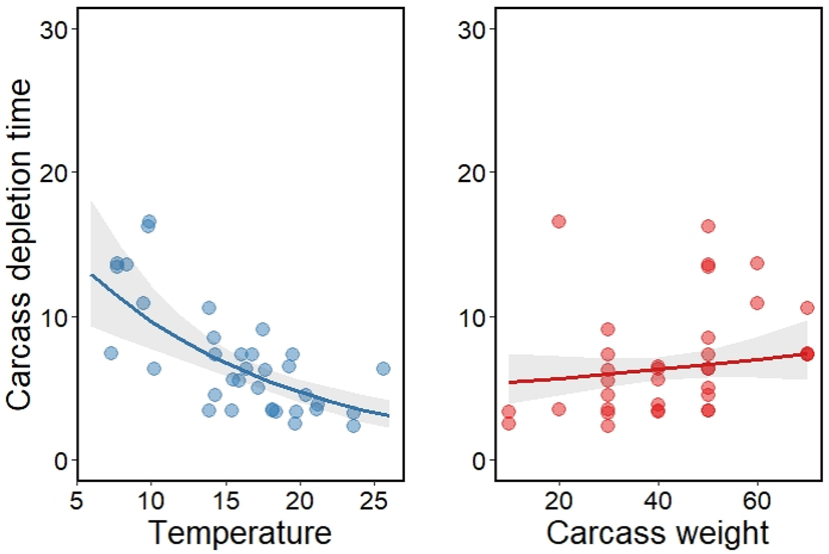
The predicted effects of temperature and carcass weight for carcass depletion time in a GLM. Points represent measured values, lines represent mean estimates, and shaded regions represent 95% confidence intervals. We calculated the relationships while keeping other independent variables constant (temperature and carcass weight are set to the mean).

The depletion times were significantly decreased by the temperature (β = − 0.072; p < 0.001, Fig. 3). The carcass weight did not significantly affect the depletion times but had a positive effect (β = 0.005; p = 0.269, Fig. 3), partially supporting hypothesis 2b.

## Discussion

### Carcass detection by scavengers

How quickly a scavenger can detect a carcass is an important aspect of their ability to acquire nutrition from carrion^6^. In our forest ecosystem most of the carcasses (88.6%) were first detected by facultative mammalian scavengers. This suggests that carcass detection by avian scavengers was likely restricted in the forest ecosystem^17^, and was apparently slower in the absence of obligate scavengers. This system was also missing large predators that kill adult ungulates, which may reduce the ability of avian scavengers to detect carcasses, as avian scavengers efficiently detect and utilize carcasses by tracking large predators^24^. However, because most mammals have good olfactory senses, they can detect carcasses themselves when searching for food even in the absence of the species that promote carcass detection.

The carcass detection ability of each species is not necessarily proportional to their scavenging frequency and instead it may depend on specific traits, dependence on nutrition from carrion, and their population density. Raccoon dogs, the most frequent scavenger, were the first scavenger at nearly half of the carcasses, while Asian black bears were the second most frequent scavenger but did not detect carcasses significantly earlier than other species. Masked palm civets and black kites, which are the least frequent scavenger^17^, also never detected carcasses first. Although raccoon dogs first detected carcasses significantly more often than other species, their detection did not lead to faster detections of other scavengers, suggesting they do not play a role in promoting carcass acquisition for the scavenger guild. This lack of species that signal the location of carrion to other species may be one of the key aspects missing in the community due to the lack of obligate scavengers. As for raccoon dogs, the population density in this study area appears higher than other species, and this may have led to their earlier carcass detections (Table S1).

We found that temperature noticeably affected the carcass detection time by vertebrate facultative scavengers. The activities of invertebrate scavengers and decomposers (fungi and microorganisms) accelerate as the temperature rises leading to more rapid carcass decomposition^1,25^. The resulting odor associated with carcass decomposition spreads, and scavengers are often able detect the carcasses more easily^8,26,27^. Our results support the results of previous studies that have shown the importance of olfactory cues and temperature to affect scavengers’ ability to find the mammalian carcass^8,26,27^. In contrast, the absence of understory vegetation had no effect on the detection time by facultative scavengers. This implies that visual cues are not as important for carcass detection in this system, which is contrary to the results of previous studies^28,29^, although the importance of visual cues often decreases in more closed habitats^30,31^. This might be because facultative mammalian scavengers put less emphasis on visual cues to detect carcasses than avian scavengers, and the richness of avian scavengers was inherently low^17^. Furthermore, the understory vegetation might also provide shelter to reduce some risks (e.g., competition with larger species, including humans).

### Carcass depletion time

Despite the lack of obligate scavengers in the system, we found that carcasses were completely consumed on average in about a week. This rank is not among the slowest compared to other studies of scavenging on ungulates where scavenging communities contained obligate scavengers. This remained even if limited to studies primarily conducted in forest ecosystems from the result that 8th depletion time among 15 studies. Together, these results suggest that our system made up entirely of facultative scavengers can still provide ecosystem services by removing carrion from the ecosystem. In our study area, this may be dependent on the most frequent scavengers (raccoon dogs and Asian black bears^17^), which were also the primary consumers of carrion. Another noteworthy point was that it was the second fastest carcass depletion time among study systems without obligate scavengers, and in many systems without obligate scavengers it takes > 1 week for ungulates to be consumed (Fig. S1). The depletion time in our system provides important data showing it had comparatively faster depletion time despite the community being composed entirely of facultative scavengers.

The carcass depletion time was strongly influenced by the temperature. Considering that carcass detection by vertebrates also appears dependent on temperature and odor, there is a tradeoff in the competitive interactions between invertebrate and vertebrate scavengers for finding and exploiting a carcass^21,22,32^. Specifically, during warmer temperatures, a facultative scavenger can detect the carcass earlier but they must compete more with invertebrates. In contrast, during colder temperatures facultative scavengers compete much less with invertebrates and can scavenge for longer durations, but they cannot detect the carcass as easily or quickly. While we did not directly measure the scavenging by invertebrates in the system, they undoubtedly play a role in carrion consumption and removal of waste from the system. Our result also showed that carcass depletion time tended to increase with carcass weight, but this was not a significant effect. This suggests that competition between vertebrate and invertebrates is a more important factor in carcass depletion. Future studies are necessary to quantify scavenging by all classes of organisms to fully understand the mechanisms of scavenger contributions to ecosystem stability.

### Conclusions

We found the facultative scavenger community, composed primarily of omnivorous mammals in our Asian temperate forest ecosystem, was capable of consuming carrion and providing ecosystem services in the system. Our results point to the importance of carrion for nutrition in mammalian carnivores, not just obligate scavengers, and highlight that facultative scavenging is widespread and fills important ecological roles (e.g., by providing ecosystem services; Ref.^1,4,5^) while also forming weak food web links (e.g., Ref.^3,16^). While we assumed the absence of obligate scavengers and in closed canopy forests could slow the detection of carcasses due to the social cues vultures provide to the community, we found that facultative scavengers nevertheless consumed carrion relatively quickly (Fig. S1). We also found that detection and consumption by facultative mammalian scavengers are sensitive to environmental factors (e.g., temperature). To understand the stability and maintenance of ecosystems, future research will need to evaluate scavenging interactions within the various biological kingdoms (i.e., vertebrates, invertebrates and microbes) and the abiotic and biotic factors that affect these interactions.

## Materials and methods

### Study area

Our research was conducted at Nikko National Park in central Japan (See Ref.^17^; approximately 1150 km^2^; 36° 36′ N–37° 05′ N, 139° 19′ E–139° 51′ E). The mean annual temperature was 7.7 °C (− 12.9 to 27.7 °C) and the mean annual rainfall was 2131 mm. The forest types included deciduous broadleaved forests (comprised mainly of *Quercus serrata*, *Q. crispula Blume*, and *Cerasus jamasakura*), conifer plantation forests (comprised mainly *Cryptomeria japonica, Chamaecyparis obtuse*, and *Larix kaempferi*), and also patchy mixed forests. The forest floor consisted primarily of bamboo grasses in each forest type, but some places had no understory vegetation due to the foraging pressure of overpopulated deer.

There is no large obligate mammalian predator in the study area, so the carrion sources of large mammals available to vertebrate scavengers are mostly natural-caused deaths (e.g., disease, starvation, and neonatal predation) or human-caused deaths (e.g., culling) rather than predation. The main mammalian scavengers at ungulate carcasses in the system are Asian black bear, wild boar (*Sus scrofa*), red fox (*Vulpes vulpes*), raccoon dog, masked palm civet, and Japanese marten (*Martes melampus*). The main avian scavengers are the jungle crow (*Corvus macrorhynchos*), black kite, and mountain hawk-eagle (*Nisaetus nipalensis*)^17^.

### Data collection

We obtained 44 fresh deer carcasses (n_summer_ = 19, n_autumn_ = 25), that had not previously been scavenged, from culled nuisance animals or animals killed through vehicle collisions from June to November in 2016 and 2017. We used the culling method that most minimizes pain and distress to the animal, in accordance with the “Welfare and Management of Animals Act” (Ministry of the Environment) and “Specified Wildlife Conservation and Management Plan” (Tochigi Prefecture 2018). We did not receive any ethical approval from the animal ethics committee of Tokyo University of Agriculture and Technology as handling of dead wild animal carcasses is not covered by the committee. We performed all handlings of the carcasses according to the guidelines of the American Society of Mammalogists^33^ and the guidelines for animal research set forth by the Mammal Society of Japan^34^.

After weighing each deer carcass (and reporting in 10-kg increments), we placed the deer carcasses at randomly selected locations where the canopy was closed and monitored with camera traps (Ltl Acorn 6210, USA) that were programmed to record 30-s videos at each trigger with a 30-s refractory period until > 80% of the carcass including bones and skins had been consumed by a scavenger (see details Ref.^17^). Each carcass was placed at a distance of > 1 km from other carcasses when we obtained the deer carcasses during the same period. In addition, the time between placing subsequent deer carcasses within a radius of 500 m was > 1 month. We secured the deer carcasses to the nearest tree using wire rope to prevent them from being removed from the view of the camera by scavengers.

We also recorded temperature (°C) and understory vegetation on the day we placed each deer carcass. We corrected the temperature at each carcass site based on the difference in altitude from the weather observatory closest to the site by subtracting or adding 0.55 °C for each 100 m increase or decrease in altitude, respectively, according to the recession ratio of temperature^35^. We classified the understory vegetation as either 1 (where the cover ratio of plants with a height just over each carcass within a 3-m radius of the carcass > 50%) or 0 (the cover ratio < 50%).

### Data analyses

We identified each vertebrate scavenger species and classified vertebrate scavengers as either mammalian or avian classes. We used program R 3.2.4^36^ and considered p-values of < 0.05 to be statistically significant for all of our statistical analyses.

To understand the pattern of carcass detection (Hypothesis 1a), we calculated the mean carcass detection time for each scavenger species. We used Wilcoxon rank sum test^37^ to test differences in the detection times between each scavenger species with all other scavengers. To identify whether there are species that detect deer carcasses earlier than other species, we first calculated the number of carcasses where each scavenger was the first to detect a carcass. We then used Fisher’s exact tests^38^ to examine differences in the proportion of first detected carcasses between the classes of mammalian and avian scavengers, and also among scavenger pairs (where we compared each scavenger species against all others).

To determine whether any key species detecting carcasses first lead to faster carcass detections of other scavengers (Hypothesis 1b), we first designated the species with the highest frequency of first detections (raccoon dogs) as the key species. We then developed a generalized linear model (GLM), using the carcass detection times for all scavengers excluding raccoon dogs and the other species with relatively few detections (masked palm civets of eight detections, mountain hawk-eagles of five detections, and black kites of four detections) as the dependent variables. The GLM was best fit to a Gamma distribution with a log link. We used the presence or absence (0 or 1) of raccoon dogs detectiong carcasses first as a independent variable in the model.

To understand the factors that determine carcass detection time for the scavenging community (Hypothesis 1c), we developed a GLM. We used the first detection times for each deer carcass as the dependent variables which were best fit to a Gamma distribution with a log link, and used temperature (°C) and the presence of understory vegetation (0 or 1) as the independent variables (Table 1).

To understand the pattern of carcass depletion time (Hypothesis 2a), we calculated the carcass depletion time for each carcass. We considered the complete carcass consumption when only bones and skin remained or when carcasses eith edible portions were removed from the camera by scavengers^18^. We used Kaplan–Meier survival analyses using the package “survival”^39^ and estimated the probability of deer carcass persistence across time. We censored the trials in which we did not monitor the carcass until it was completely consumed (n = 9) due to camera malfunctions or displacement by Asian black bears. We also compared our mean depletion time to the mean depletion times reported by Ref.^18^ for studies using ungulates that weighed < 100 kg. We classified studies conducted in forest and non-forest areas using Google Maps (Google LLC, USA) and reported latitude and longitude or the study site in the original paper.

To evaluate the factors that affect the carcass depletion time (Hypothesis 2b), we developed a GLM. We used the carcass depletion time as the dependent variable, which was best fit to a Gamma distribution with a log link. We used the independent variables temperature (°C) and carcass weight (kg), in the model.

## Supplementary Information

Supplementary Information.

## Data Availability

The data for this manuscript will be submitted to the Illinois Data Bank (https://databank.illinois.edu/).
