# Supplementary material for: Carcass detection and consumption by facultative scavengers in forest ecosystem highlights the value of their ecosystem services
Source: Sci Rep. 2022 Sep 30;12:16451. doi: 10.1038/s41598-022-20465-4 (PMC9525280; doi:10.1038/s41598-022-20465-4)
Supplement: Supplementary file 1 — Supplementary Information. [file 41598_2022_20465_MOESM1_ESM.docx]

**Supplementary Information**

**Figure S1.** A comparison of carcass depletion times for ungulates weighing <100 kg from Sebastián-González et al. (2020). The mean carcass depletion time (days) for each study is shown, with study areas in forest area shown in green, other area shown in blue and our study in red. The asterisks shown the systems without obligate scavengers.

**Literature Cited**

1. Allen, M.L., L.M. Elbroch, C.C. Wilmers, and H.U. Wittmer. 2014. Trophic facilitation or limitation? Comparative effects of pumas and black bears on the scavenger community. PLoS One 9: e102257.
2. Allen, M.L., L.M. Elbroch, C.C. Wilmers, and H.U. Wittmer. 2015. The comparative effects of large carnivores on the acquisition of carrion by scavengers. American Naturalist 185: 822–833.
3. Krofel M. 2011. Monitoring of facultative avian scavengers on large mammal carcasses in Dinaric forest of Slovenia. Acrocephalus 32: 45−51.
4. Krofel, M., Kos, I., & Jerina, K. (2012). The noble cats and the big bad scavengers: effects of dominant scavengers on solitary predators. *Behavioral Ecology and Sociobiology*, *66*(9), 1297-1304.
5. Mateo-Tomás P, Olea PP, Moleón M, Vicente J, Botella F, Selva N, Viñuela,J & Sánchez-Zapata JA. 2015. From regional to global patterns in vertebrate scavenger communities subsidized by big game hunting. Diversity and Distributions 21: 913-924.
6. Moleón M, Sánchez-Zapata JA, Sebastián-González E, Owen-Smith N. 2015. Carcass size shapes the structure and functioning of an African scavenging assemblage. Oikos, 124: 1391–1403.
7. Morales-Reyes Z, Martín-López B, Moleón M, Mateo-Tomás P, Olea PP, Arrondo E, Donázar JA & Sánchez-Zapata JA. 2019. Shepherds’ local knowledge and scientific data on the scavenging ecosystem service: insights for conservation. Ambio 48: 48–60.
8. Perrig P.L., S.A. Lambertucci, E. Donadio, J. Padro, J.N. Pauli. 2019. Monitoring vultures in the 21st century: the need for standarized protocols. Journal of Applied Ecology 56:796-801.
9. Sebastián-González, E., Z. Morales-Reyes, F. Botella, L. Naves-Alegre, J.M. Pérez-García, P. Mateo-Tomás, P.P. Olea, M. Moleón, J.M. Barbosa, F. Hiraldo, E. Arrondo, J.A. Donázar, A. Cortés-Avizanda, N. Selva, S.A. Lambertucci, A. Bhattacharjee, A. Brewer, J.D. Anadón, E. Abernethy, K. Turner, J.C. Beasley, T.L. DeVault, H.C. Gerke, O.E. Rhodes Jr, A. Ordiz, C. Wikenros, B. Zimmermann, P. Wabakken, C.C. Wilmers, J.A. Smith, C.J. Kendall, D. Ogada, E. Frehner, M.L. Allen, H.U. Wittmer, J.R.A. Butler, J.T. du Toit, A. Margalida, P. Oliva-Vidal, D. Wilson, K. Jerina, M. Krofel, R. Kostecke, R. Inger, E. Per, Y. Ayhan, H. Ulosoy, D. Vural, A. Inagaki, S. Koike, A. Samson, P.L. Perrig, E. Spencer, T.M. Newsome, M. Heurich, J.D. Anadon, E.R. Buechley, and J.A. Sánchez-Zapata. 2020. Network structure of vertebrate scavenger assemblages is driven by ecosystem productivity and human impact at a global scale. Ecography, 43(8), 1143-1155.
10. Turner KL, Abernethy EF, Conner LM, Rhodes OE Jr & Beasley JC. 2017. Abiotic and biotic factors modulate carrion fate and vertebrate scavenging communities. Ecology 98: 2413–2424.
11. Wikenros C, Sand H, Ahlqvist P, Liberg O. 2013. Biomass Flow and Scavengers Use of Carcasses after Re-Colonization of an Apex Predator. PLoS ONE 8(10): e77373.

**Table S1.** Relative Abundance Index (RAI) for each mammal scavenger species in the study area. We randomly set 20 camera traps (images, capture interval=1 minute) from June 1 to December 15, 2017. One or two cameras were in each 5 km mesh, and each camera was a distance of at least 500 m radius from each deer carcass. A total of 1328 images were captured during 3786 camera days; we calculated the RAI (Equation) for each scavenger species.

RAI = (total number of individuals) ⁄ (camera days)

| Common name | Species | RAI |
| --- | --- | --- |
| Asian black bear | *Ursus thibetanus* | 0.055 |
| Wild boar | *Sus scrofa* | 0.080 |
| Raccoon dog | *Nyctereutes procyonoides* | 0.155 |
| Red fox | *Vulpes vulpes* | 0.006 |
| Japanese marten | *Martes melampus* | 0.040 |
| Masked musang | *Paguma larvata* | 0.016 |
